# First person – Johann Maass

**DOI:** 10.1242/dmm.052653

**Published:** 2025-09-22

## Abstract

First Person is a series of interviews with the first authors of a selection of papers published in Disease Models & Mechanisms, helping researchers promote themselves alongside their papers. Johann Maass is first author on ‘ Models of Bosch-Boonstra-Schaaf optic atrophy syndrome reveal genotype-phenotype correlations in brain structure and behavior’, published in DMM. Johann conducted the research described in this article while a medical doctoral candidate in Christian P. Schaaf's lab at Heidelberg University Clinic, Heidelberg, Germany. He is now a medical student and research intern in the lab of Timothy Yu at Boston Children's Hospital, Boston, MA, USA, investigating advancements in gene therapy for patients with debilitating neurological diseases.


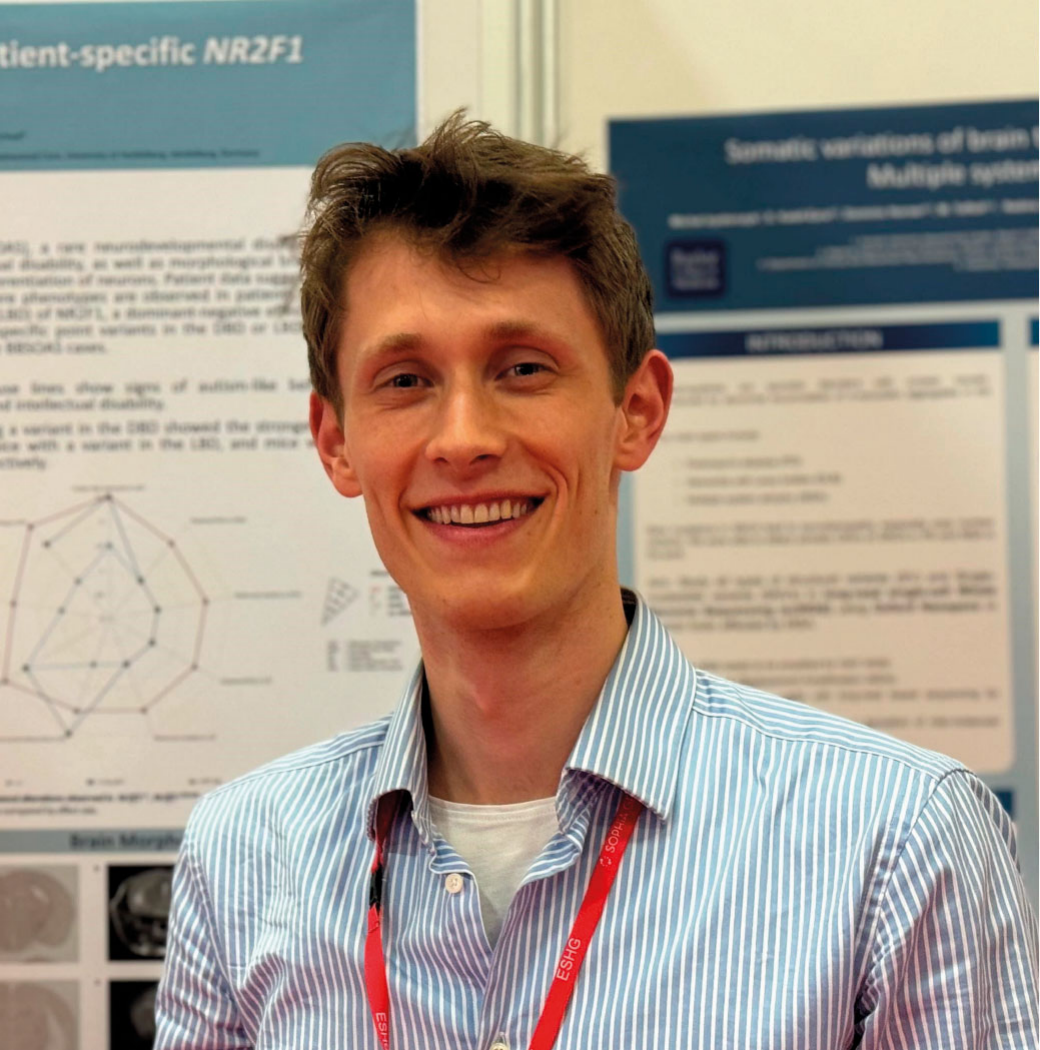


**Johann Maass**

**Who or what inspired you to become a scientist?**

High-school biology and mathematics classes. The progress of what we knew 30 years ago versus what we know now is very inspiring to me.

**What is the main question or challenge in disease biology you are addressing in this paper? How did you go about investigating your question or challenge?**

The mouse models previously available for Bosch-Boonstra-Schaaf optic atrophy syndrome (BBSOAS) did not adequately recapitulate core symptoms of the disorder. If a model lacks these key features, it becomes difficult to focus on, analyse and potentially treat the corresponding symptoms. We also know that BBSOAS presents with a wide phenotypic spectrum, ranging from mild to severe, likely based on genotype-phenotype correlations. Importantly, the earlier mouse models genetically corresponded to patients with relatively mild manifestations of the disease. To address this limitation, we generated two additional mouse models carrying patient-specific variants expected to represent the more severe end of the spectrum. Once established, we again asked the critical question: how well do these models capture the core symptoms of BBSOAS? To evaluate this, we employed an extensive behavioural test battery alongside detailed brain morphology analyses. Encouragingly, we observed a striking face validity between our new models and the human phenotype.

We generated mouse models with genetic modifications that very accurately recapitulate the core symptoms of BBSOAS seen in patients

**How would you explain the main findings of your paper to non-scientific family and friends?**

We generated mouse models with genetic modifications that very accurately recapitulate the core symptoms of BBSOAS seen in patients.

**What are the potential implications of these results for disease biology and the possible impact on patients?**

We now have a mouse model that allows us to analyse the disease in great detail and potentially test therapeutic approaches. Having established that mice with a deletion display a less severe phenotype than those carrying a missense variant in a specific domain, we present a strong case for exploring allele-selective knockdown strategies, such as antisense oligonucleotides. This approach could alleviate the phenotype and potentially provide the first causal therapeutic strategy for BBSOAS.

**Figure:**
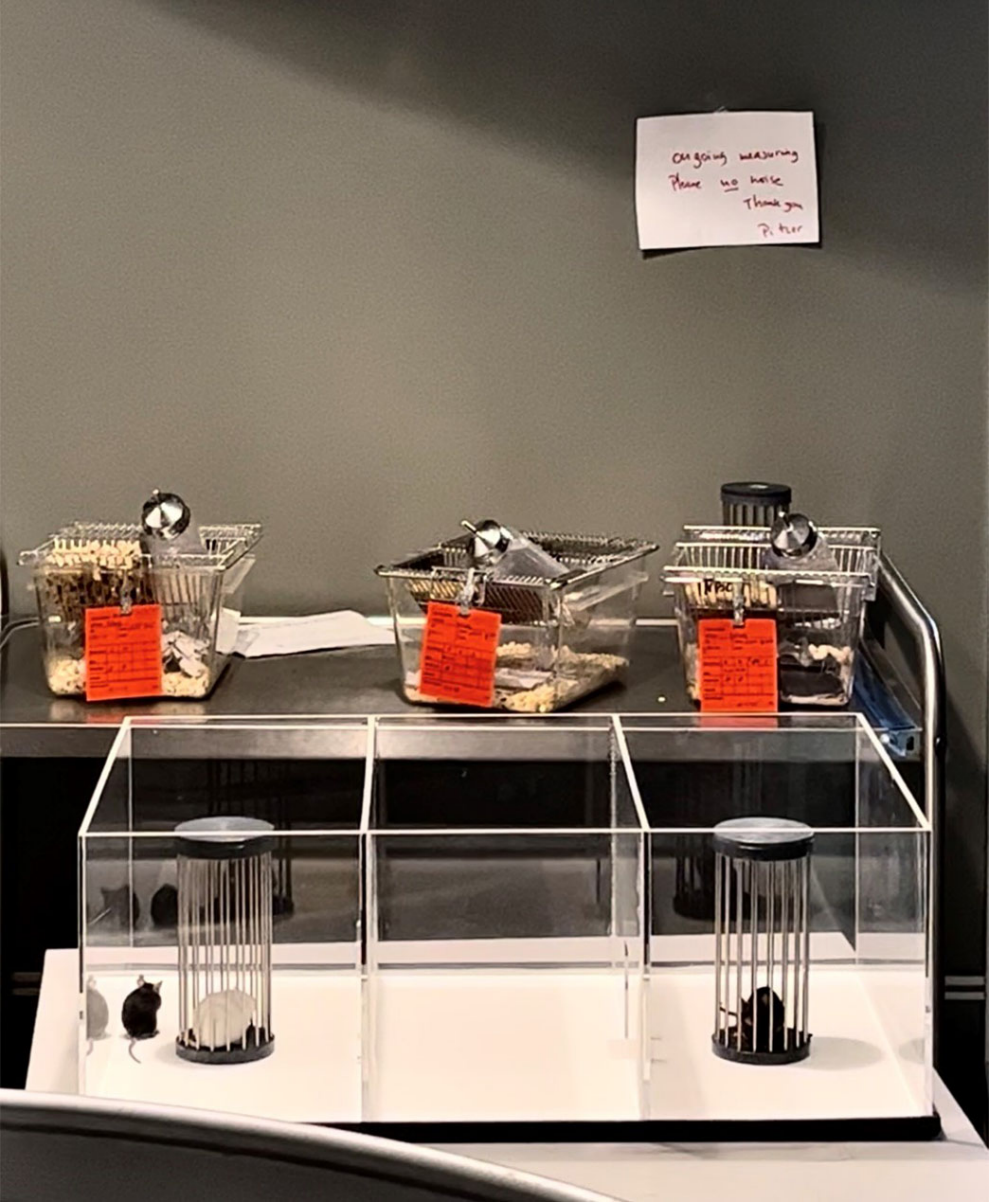
Final test run of the social interaction test (analysing social preferences of mice) to calibrate the system prior to the beginning of the test battery.

**Why did you choose DMM for your paper?**

As this is only my second first author publication, we wanted to have a journal that advocates and helps with great scientific quality. DMM has a reputation of being very thorough in their editorial review.

It is hardly ever an experiment or a protocol that is responsible for major delays

**Given your current role, what challenges do you face and what changes could improve the professional lives of other scientists in this role?**

It is hardly ever an experiment or a protocol that is responsible for major delays. Most of the time is lost waiting for approvals or gathering research equipment. I believe that planning well ahead is the most important advice one can follow – although I must admit, I am not particularly good at it myself.

**What's next for you?**

Once I return from Boston to Germany, I plan to complete my medical doctoral thesis and finish my medical education to become a physician. My goal is to work as a clinician-scientist and help bridge the gap from bench to bedside. The work presented in this paper lays the foundation for even more exciting studies to come, bringing us a step closer to delivering individualized therapies.

**Tell us something interesting about yourself that wouldn't be on your CV**

I prioritize family before work and creative freedom at work before salary.

**What is the reason for you pursuing academia and not business or finance?**

A lot of very intelligent people in my close circle are considering going into finance or business in general. Sometimes it is a decision for more money, but more often it is a decision against the stereotype of a hard and slow academic career path. I believe much of the bad reputation of science is self-inflicted, and scientists should be more outspoken about the various benefits of their career. The freedom of choosing what to work on, whom to work with, as well as the international community are things that I value about the job perspective.
